# Indications, Causes, and Patient Risk Factors for Revision After Total Ankle Arthroplasty: A Descriptive Cross-Registry Analysis of the NJR, AOANJRR, and SwedAnkle Registries

**DOI:** 10.3390/clinpract16070133

**Published:** 2026-07-17

**Authors:** Sedeek Mosaid, Yousif Jihad, Mostafa Jihad, Ashok Marudanayagam, Paul Lee

**Affiliations:** 1 United Lincolnshire Teaching Hospitals NHS Trust, Lincoln County Hospital, Greetwell Road, Lincoln, Lincolnshire LN2 5QY, UK; 2 Royal Preston Hospital, Lancashire Teaching Hospitals NHS Foundation Trust, Sharoe Green Lane, Fulwood, Preston, Lancashire PR2 9HT, UK

**Keywords:** total ankle arthroplasty, total ankle replacement, joint registry, revision arthroplasty, survivorship, risk factors, aseptic loosening, cross-registry analysis, registry harmonisation

## Abstract

**Background/Objectives:** Total ankle arthroplasty (TAA) is increasingly used to treat end-stage ankle arthritis, but comprehensive cross-registry data on revision patterns remain limited. This study describes indications, causes, and patient risk factors for revisions using three national registries. **Methods:** Aggregate data were extracted from the UK National Joint Registry (NJR; 22nd Annual Report 2025; *n* = 11,321), the Australian Orthopaedic Association National Joint Replacement Registry (AOANJRR; 2025 Ankle Supplementary Report; *n* = 5379), and the Swedish Ankle Registry (SwedAnkle; Annual Report 2024; *n* = 1852; survival estimates from a published sub-cohort, *n* = 1226). Metrics were compared descriptively without inferential pooling. **Results:** Across registries, 18,552 primary TAAs were identified. Ten-year cumulative per cent revision (CPR) was 9.54% (95% confidence interval [CI], 8.75–10.39) in the NJR, 13.5% (95% CI, 12.1–15.1) in the AOANJRR osteoarthritis sub-cohort, and approximately 26% (author-derived from Kaplan–Meier curves; see Methods) in SwedAnkle (1993 onwards implants). Aseptic loosening was the predominant cause of revision in all three registries; the rank of subsequent causes differed between registries (infection was second in the NJR and AOANJRR; in SwedAnkle, infection ranked below insert wear/breakage at 11.5%). AOANJRR Cox analysis identified younger age (hazard ratios (HRs), 2.00 for <55 vs. ≥75 years; 95% CI, 1.30–3.07; *p* = 0.001), earlier surgical era (HR, 1.91 for pre-2015 vs. 2015–2024; 95% CI, 1.53–2.38; *p* < 0.001), and obesity (HR, 1.52 for body mass index (BMI) ≥ 30; 95% CI, 1.06–2.19; *p* = 0.023) as significant independent predictors in AOANJRR Cox proportional hazards models (osteoarthritis sub-cohort for age, sex, BMI and ASA; all-diagnoses primary cohort for surgical era). Sex and American Society of Anesthesiologists (ASA) scores were not significant. **Conclusions:** Aseptic loosening was the predominant cause of revision in all three registries. The rank of subsequent causes differed: in the NJR and AOANJRR, infection was the second most frequent cause; in SwedAnkle, infection ranked below insert wear/breakage. Younger age, obesity, and earlier surgical era were independent predictors of revision in AOANJRR Cox proportional hazards models. The post-2015 era was associated with approximately 48% lower revision hazard; this association cannot be interpreted causally, as the independent contributions of implant design, surgical technique, patient selection, and other secular changes cannot be isolated from registry data. These findings may inform preoperative counselling, implant selection, and registry harmonisation efforts.

## 1. Introduction

End-stage ankle arthritis is a debilitating condition, and affected patients experience functional disability comparable to end-stage hip arthrosis [1]. The two principal surgical treatments are ankle arthrodesis (fusion) and total ankle arthroplasty (TAA). While arthrodesis abolishes tibiotalar motion and may increase mechanical stress on adjacent hindfoot joints [2], recent large national-population and comparative studies have not consistently shown a reduction in subsequent adjacent joint surgery following TAA [3,4].

TAA utilisation has risen steadily worldwide [5]. However, the procedure remains associated with higher revision rates than hip or knee arthroplasty [6,7], and the indications and risk factors for revision are incompletely characterised at the international level [5,8,9].

Three of the best-established registries with ankle-specific data are the UK National Joint Registry (NJR), the Australian Orthopaedic Association National Joint Replacement Registry (AOANJRR), and the Swedish Ankle Registry (SwedAnkle). Each employs distinct methodologies for data collection, endpoint definition, and statistical reporting, limiting direct statistical pooling [10,11,12]. To our knowledge, no standardised side-by-side synthesis integrating revision data from these three registries has been published. However, the International Ankle Arthroplasty Registry Consortium has begun to address cross-registry methodology more broadly [5,9]. This study provides a comprehensive descriptive analysis across the NJR, AOANJRR, and SwedAnkle, characterising primary TAA volume, overall implant survivorship, causes and types of revision, and patient-related revision risk factors.

## 2. Materials and Methods

### 2.1. Study Design and Data Sources

This was a secondary, descriptive analysis of publicly available aggregate data from three national registries (Table 1): the NJR (22nd Annual Report 2025; England and Wales, 1 January 2010 to 31 December 2024; *n* = 11,321; cases from Northern Ireland, Guernsey, and the Isle of Man are not currently included in the NJR’s ankle survivorship analyses because of differences in data linkage and audit coverage) [10]; AOANJRR (2025 Ankle Supplementary Report; 2006 to 31 December 2024; *n* = 5379, with osteoarthritis (OA) sub-cohort *n* = 5079) [11]; and SwedAnkle (Annual Report 2024; 1993–2024; *n* = 1852) [12,13]. No individual patient-level data were accessed.

Kaplan–Meier (KM) estimates and Cox proportional hazards ratios were extracted directly from registry reports or, where survival fractions were reported, were transformed by the authors into cumulative per cent revision (CPR = 100 × (1 − survival fraction)); author-derived values are labelled in Table 2. Risk factor analyses (age, sex, body mass index [BMI], and American Society of Anesthesiologists [ASA] score) are reported by the AOANJRR for its OA sub-cohort (*n* = 5079; Tables A16–A19 [11]), while the surgical-era hazard ratio (pre-2015 vs. 2015–2024) is reported for the all-diagnoses primary cohort (*n* = 5379; Table A14, Figure A7 [11]). The NJR and SwedAnkle do not publish equivalent multivariable models; therefore, risk factor findings are exclusively derived from the AOANJRR.

#### Extraction of Figure-Derived SwedAnkle Values

SwedAnkle does not publish numerical CPR at fixed time points; survival probability is shown only as Kaplan–Meier curves by Undén et al. (2020) [14]. The four values used in Table 2 (CPR at 5, 10, 15, and 20 years) were read from these curves and converted to CPR using the equation above. Readings were calibrated against the printed year and survival probability axes of the source figure, with a precision of approximately ±1–2 percentage points. The extraction procedure was performed manually using two independent readings of each time point, with the average value reported; the procedure is described in full to permit reproduction by interested readers. The resulting 10-year CPR of ~26% is consistent with Henricson et al. (2011) [13], who reported a 10-year survival of 0.69 (95% CI, 0.67–0.71; CPR ~31%) for the earlier 780-case SwedAnkle cohort. These four values are highlighted with asterisks in Table 2; all other results in this analysis come from tabulated registry sources (NJR Table 3.A3 [10]; AOANJRR Tables A11, A14, A16–A19 [11]).

### 2.2. Definitions and Metric Differences

The three registries use non-commensurate metrics (Table 1). The NJR reports cause-specific revision as incidence rates per 100 prosthesis-years and overall survivorship as KM CPR with 95% CIs; the AOANJRR reports causes as percentages of all revisions and provides Cox hazard ratios (HRs); SwedAnkle reports causes as percentages and survivorship via KM curves. Importantly, isolated meniscus (polyethylene insert) exchange is tracked by SwedAnkle but excluded from the formal KM survival endpoint, consistent with Undén et al. [14]. The NJR and AOANJRR include insert exchanges in their revision counts. These differences preclude inferential pooling; all cross-registry comparisons are descriptive only. Throughout the registries, ‘TAA’ is used as the primary term; the NJR uses ‘ankle replacement’ and the AOANJRR uses ‘ankle arthroplasty’ interchangeably for the same procedure.

### 2.3. Reporting Standards

Reporting follows the STROBE [15] and RECORD [16] statements where applicable to aggregate registry data. As all data were derived from publicly available, de-identified registry reports, formal ethical approval and patient consent were not required.

## 3. Results

### 3.1. Cohort Overview and Volume Trends

Across the three registries, 18,552 primary TAAs were captured (Table 1), comprising 11,321 (61.0%) from the NJR, 5379 (29.0%) from the AOANJRR, and 1852 (10.0%) from SwedAnkle. For SwedAnkle, the full cohort of 1852 prostheses (1993–2024) was used for indication and demographic analyses; survival estimates are based on the Undén et al. (2020) [14] sub-cohort of 1226 prostheses operated through 2016 (see Section 3.2). The median age was 69 years (NJR) and 68 years (AOANJRR); SwedAnkle reported approximately 64 years for the 2024 calendar-year cohort only (these age metrics are not directly comparable across registries). Males comprised the majority (59–62%) of patients in all registries [10,11,12].

### 3.2. Overall Survivorship

CPR at standard time points is presented in Table 2. The NJR, which predominantly captures modern designs, reported the lowest revision rates (10-year CPR 9.54%, 95% CI, 8.75–10.39) [10]. The AOANJRR demonstrated substantially lower revision rates in the modern 2015–2024 era than in the pre-2015 era; the all-eras OA sub-cohort’s 10-year CPR was 13.5% (95% CI, 12.1–15.1%; AOANJRR Table A11) [11]. SwedAnkle, which includes procedures from 1993, showed higher long-term estimates: the Undén et al. (2020) cohort of 1226 prostheses had a 10-year survival fraction of approximately 0.74 overall (author-derived CPR ~26%), while the more recently introduced design subgroup 10-year survival fraction was approximately 0.84 (author-derived CPR ~16%) [14]. An earlier registry-wide analysis of 780 cases (designs to 2010; Henricson et al., 2011) reported a 10-year survival fraction of 0.69 (95% CI, 0.67–0.71) [13].

Substantial variation across registries reflects differences in cohort composition, particularly the implant era, rather than directly comparable differences in surgical outcomes between countries. SwedAnkle includes early-generation designs (e.g., STAR [Scandinavian Total Ankle Replacement], AES [Ankle Evolutive System], BP [Buechel–Pappas]) known to carry higher revision rates [12,14].

### 3.3. Causes of Revision

Aseptic loosening was the dominant indication across all registries (Table 3). Within the NJR, the primary reasons for revision (not mutually exclusive) occurred at the following rates per 100 prosthesis-years: aseptic loosening (0.43), infection (0.27), lysis (0.22), and malalignment (0.19) [10]. Among the 424 first revisions of known primary procedures in the AOANJRR, the most frequent indications were aseptic loosening (33.0%, *n* = 140), infection (14.4%, *n* = 61), lysis (9.4%, *n* = 40), insert breakage (9.0%, *n* = 38), instability (8.0%, *n* = 34), and malalignment (1.7%, *n* = 7) [11]. The remaining 24.5% (*n* = 104) of AOANJRR first revisions were recorded under other indications, including pain, periprosthetic fracture, dislocation, wear, and unspecified categories [11]. For SwedAnkle (*n* = 355 cumulative revisions, 1993–2024) the reported revision rates were aseptic loosening, 47.6% (*n* = 169); insert wear/breakage, 11.5% (*n* = 41); infection, 9.6% (*n* = 34); unexplained pain, 8.2% (*n* = 29); technical error, 6.5% (*n* = 23); malalignment, 6.2% (*n* = 22); instability 4.8%, (*n* = 17); and periprosthetic fracture, 3.7% (*n* = 13). SwedAnkle does not separate isolated osteolysis as a distinct revision indication; lysis-driven failures are typically captured under aseptic loosening [12].

### 3.4. Type of Revision Surgery

The AOANJRR provides detailed revision-type data (Table 4). Of 424 revisions, the most common procedure was insert-only exchange (44.8%), followed by tibial/talar revision (19.3%), conversion to arthrodesis (11.6%), tibial-only (9.2%), and talar-only (7.1%). Less common procedures included cement spacer insertion (4.7%), prosthesis removal (1.7%), and minor component revision (1.7%) [11]. The 11.6% conversion-to-arthrodesis rate is clinically important because salvage arthrodesis after a failed TAA is not equivalent in outcome to primary ankle arthrodesis. A recent systematic review reported a non-union rate of 13% for conversion of TAA to fusion, together with substantial variation in surgical technique reflecting the need to address prior bone loss, typically with bulk allograft or extended fixation [8].

### 3.5. Age as a Predictor of Revision

Younger age was a statistically significant independent predictor of revision (AOANJRR HR, 2.00 for <55 vs. ≥75 years; 95% CI, 1.30–3.07; *p* = 0.001) [11]. Unadjusted 10-year CPR varied across age groups (<55 = 14.5%; 55–64 = 19.1%; 65–74 = 11.8%; ≥75 = 7.2%; Table 5); the higher rate in the 55–64 group most likely reflects cohort composition (including a higher proportion of pre-2015 implants in this age band) rather than a true reversal of the age–revision relationship. NJR sex-stratified analysis revealed a marked age–sex pattern (Table 6): females aged <65 years had the highest 7-year CPR (11.52%; 95% CI, 9.73–13.61), whereas females aged ≥75 years had the lowest (2.05%; 95% CI, 1.26–3.33). Males showed a less pronounced age gradient (8.53% for <65 vs. 3.71% for ≥75 years) [10].

### 3.6. BMI and ASA Score

Obesity (BMI ≥ 30 kg/m^2^) was a significant predictor of revision (AOANJRR HR, 1.52; 95% CI, 1.06–2.19; *p* = 0.023; Table 7) [11]. The AOANJRR reports BMI-stratified CPR data only up to the 7-year time point. The ASA score was not associated with revision: ASA 3 vs. ASA 1 HR, 1.12 (95% CI, 0.65–1.96; *p* = 0.678); ASA 2 vs. ASA 1 HR, 1.28 (95% CI, 0.77–2.13; *p* = 0.332). The point estimate for ASA 2 was marginally higher than that for ASA 3, but both estimates are non-significant with widely overlapping CIs, and no clinical inference should be drawn from this [11]. Comparable BMI and ASA risk factor data are not reported by the NJR or SwedAnkle.

### 3.7. Temporal Improvement and Implant Generations

A significant improvement in TAA survivorship was observed over time. Procedures performed pre-2015 had an HR for revision of 1.91 (95% CI, 1.53–2.38; *p* < 0.001) compared with 2015–2024 (Table 8; AOANJRR all-diagnoses primary cohort, *n* = 5379, Table A14, adjusted for age and sex), representing an approximately 48% reduction in revision hazard in the modern era (reciprocal of the reported HR) [11]. SwedAnkle data corroborate this trend: within the Undén et al. (2020) cohort, as early-generation designs (STAR, AES, BP) had substantially higher cumulative revision proportions than more recently introduced designs [12,14].

### 3.8. Sex as a Predictor

Sex was not statistically significant as an independent predictor of revision in the AOANJRR analysis (HR, 1.22; 95% CI, 0.99–1.50; *p* = 0.063; Table 8), though the estimate is borderline, and a true effect cannot be excluded [11]. The NJR data show age-dependent variation in sex-related risk (see Section 3.5 and Table 6) [10].

### 3.9. Summary of All Predictors

**Figure 1:**
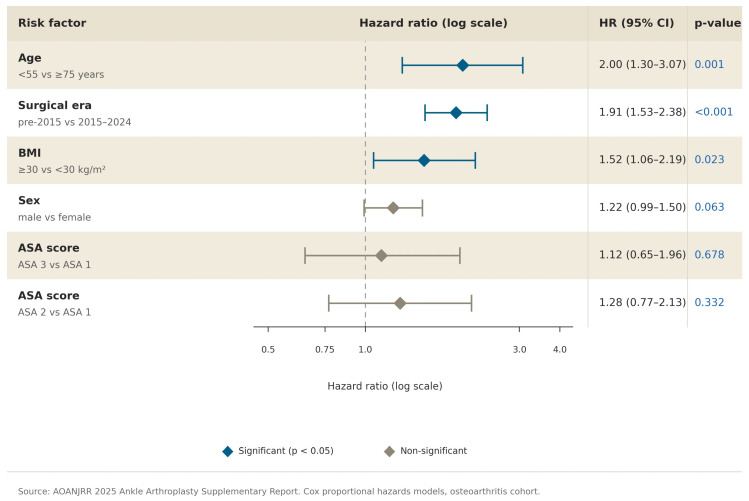
Adjusted HRs with 95% confidence intervals (CIs) for independent predictors of revision after primary TAA (AOANJRR Cox proportional hazards models; age, sex, BMI, and ASA HRs from the osteoarthritis sub-cohort (*n* = 5079, Tables A16–A19); surgical-era HR from the all-diagnoses primary cohort (*n* = 5379, Table A14, Figure A7)). Diamonds = point estimates; bars = 95% CIs; dashed line = HR, 1.00 (no effect). Blue = statistically significant (*p* < 0.05); grey = non-significant. Source: AOANJRR 2025 Ankle Supplementary Report [11].

Table 8 and Figure 1 (forest plot) summarise all independent predictors of revision from the AOANJRR Cox proportional hazards models, drawn from two separate analyses: age, sex, BMI and ASA from the osteoarthritis sub-cohort (*n* = 5079), and surgical era from the all-diagnoses primary cohort (*n* = 5379). Three predictors were statistically significant: younger age, historical surgical era, and obesity. Sex was borderline significant (HR, 1.22; 95% CI, 0.99–1.50; *p* = 0.063), and ASA score was not associated with revision risk [11]. These AOANJRR-derived risk factor estimates have not been validated against equivalent NJR or SwedAnkle multivariable models, as those registries do not publish comparable analyses.

## 4. Discussion

This cross-registry descriptive analysis of over 18,000 primary TAAs from three national registries shows four main findings. First, overall 10-year revision rates range from approximately 10% to 26%, depending on the registry and the implant generation captured. Second, aseptic loosening is the predominant cause of revision across all registries. Third, younger patient age, obesity, and historical surgical era are significant predictors of revision in analyses reported by the AOANJRR. Fourth, more recent operative eras are associated with lower reported revision rates.

### 4.1. Survivorship in Context

The NJR 10-year CPR of 9.54% is the most precisely estimated figure among the three registries, reflecting its large sample and the predominance of modern devices; however, the NJR explicitly acknowledges under-capture of conversion to arthrodesis and amputation, endpoints that would otherwise inflate the reported rate [10]. The AOANJRR shows higher pre-2015 era rates (7-year CPR 12.9%) than modern era rates (6.4%); the all-eras OA sub-cohort 10-year CPR is 13.5% (95% CI, 12.1–15.1%; Table A11), positioned between the contemporary NJR estimate and the historical SwedAnkle estimates [11]. SwedAnkle shows the highest revision estimates, reflecting the inclusion of procedures from 1993, using early-generation devices [12,13,14].

A meta-analysis by van der Plaat and colleagues reported an expected 10-year revision rate of approximately 22% [6], though this pooled estimate included historical implant cohorts. Importantly, Jennison et al. (2023), using NJR–NHS Digital data linkage, demonstrated that approximately one-third of ankle arthroplasty failures are not captured by the NJR (true 5-year survival 90.2% vs. 93.5% from NJR data alone) [17]. NJR-derived estimates should therefore be interpreted as likely underestimates of true failure rates. Importantly, the registries differ in their revision definitions (notably the SwedAnkle exclusion of isolated insert exchange, which is included by the NJR and AOANJRR), the implant generations they cover (SwedAnkle from 1993 onwards, including early-generation designs; NJR and AOANJRR predominantly capturing modern designs), follow-up windows, and statistical methods. Direct numerical comparison of revision rates between registries is therefore precluded, and all cross-registry comparisons in this manuscript are descriptive only, identifying qualitative consistencies (such as the predominance of aseptic loosening) rather than asserting numerical equivalence.

A specific and important source of systematic bias merits emphasis: SwedAnkle explicitly excludes isolated polyethylene insert (“en passant” meniscus) exchange from its Kaplan–Meier survival endpoint, counting a revision only when at least one metallic component is removed or exchanged [14]. Because the NJR and AOANJRR included isolated insert exchange within their revision definitions, the SwedAnkle survival curves are systematically and artificially superior to the British and Australian curves. Full patient-level recalibration—which would require reclassifying each excluded insert exchange as a revision event and re-fitting the Kaplan–Meier estimator—is not feasible from the aggregate published data available in the three registry reports. However, the plausible magnitude of the bias can be estimated. In the AOANJRR, insert-only exchange is the single most common revision type, comprising 44.8% of all revisions (190 of 424) [11]. If the AOANJRR proportion were applied to SwedAnkle, isolated insert exchanges currently excluded from the endpoint would represent a substantial additional volume of revision events, plausibly raising the 10-year cumulative per cent revision from approximately 26% to the mid-thirties, thereby narrowing the apparent gap with the AOANJRR osteoarthritis sub-cohort estimate of 13.5% considerably. This estimate is illustrative, not definitive, and clinical practice with respect to isolated insert exchange may itself differ between countries. The key inference for readers is directional: cross-registry comparisons involving SwedAnkle should be interpreted with the understanding that its published survival estimates represent a lower bound on the true revision rate as defined by the NJR and AOANJRR.

A further quantitative caveat applies to the NJR estimates. Jennison et al. (2023), using linked NJR–NHS Digital data, demonstrated that approximately one-third of true ankle arthroplasty failures in England are not captured by the NJR alone [17]. NJR-derived cumulative per cent revision figures reported in this manuscript should therefore be interpreted as lower bounds, with a plausible true failure rate materially higher than the reported 10-year CPR of 9.54%.

In addition to these methodological considerations, coverage limitations should moderate the interpretation of our findings. The three registries analysed here, while representative of well-established national data systems, do not capture the majority of total ankle arthroplasties performed globally each year; the prosthetic designs implanted, the surgical volumes per centre, and the thresholds for revision vary widely between countries and health systems. Prior work by the International Ankle Arthroplasty Registry Consortium has documented substantial geographical variation in national reported revision rates and surgical practice [5,9], and the qualitative patterns reported here (aseptic loosening as the predominant cause; improving survivorship with newer implant generations) should not be automatically extrapolated to registries or health systems not analysed.

### 4.2. Causes of Revision in Context

The predominance of aseptic loosening across all registries is consistent with published systematic reviews [6,8], underscoring the need for continued improvements in bone–implant fixation and wear characteristics. Infection was a leading indication across all three registries. It was the second most common indication in the AOANJRR (14.4%) and ranked third in SwedAnkle (9.6%, behind insert wear/breakage at 11.5%); the NJR reported infection at 0.27 per 100 prosthesis-years [18]. Lysis was the third most common AOANJRR indication (9.4%), highlighting the role of osteolysis, likely driven by polyethylene wear debris [19]. AOANJRR data on revision type are particularly informative: the high proportion of insert-only exchanges (44.8%) suggests many revisions are managed without major component revision; however, 11.6% resulted in conversion to arthrodesis and 1.7% in prosthesis removal [11]. Kamrad et al. (2015) reported poor survival after broader component exchange [20], raising inferential concerns about insert-only durability that warrant further registry-based comparative analysis.

### 4.3. Risk Factors

The twofold revision hazard for patients aged <55 versus ≥75 years (AOANJRR HR, 2.00) likely reflects higher activity levels and longer expected implant service life in younger patients. NJR data additionally revealed that young females (<65 years) had a particularly elevated 7-year CPR (11.52%), a pattern warranting further investigation [10]. These findings align with international evidence: Subramanian et al. (2024) reported adjusted HRs of 1.725 for patients aged <55 and 1.812 for those 55–64 in a Korean cohort of 5619 TAAs (*p* < 0.001 for both) [21], and Suh et al. (2021) confirmed younger age and elevated BMI as significant risk factors in a separate Korean cohort of 2914 TAAs [22].

Obesity (BMI ≥ 30 kg/m^2^) was a significant predictor (AOANJRR HR, 1.52) [11], supporting consideration of weight management in preoperative care. Schipper et al. (2016) reported a directionally consistent 5-year HR of 3.73 in an older single-institution obese TAA cohort [23], while Kim et al. (2023) found no significant association with complication rates at midterm follow-up in a single-institution study (*n* = 1093). Obese patients did report worse function and quality-of-life scores [24]. Sex did not reach significance overall (HR, 1.22; 95% CI, 0.99–1.50; *p* = 0.063, borderline), and ASA score was not associated with revision [11].

### 4.4. Temporal Improvement

The approximately 48% reduction in revision hazard for procedures performed from 2015 onwards (calculated as the reciprocal of the AOANJRR-reported HR, 1.91; *p* < 0.001) [11] likely reflects newer implant designs, evolving surgical technique, and changes in patient selection. The independent contribution of each cannot be isolated using registry data. Within the AOANJRR OA cohort, implant design characteristics were significantly associated with differences in survival outcomes. Fixed-bearing inserts demonstrated markedly lower revision rates than mobile-bearing designs (HR, 3.20 for mobile vs. fixed beyond 9 months; 95% CI, 2.35–4.35; *p* < 0.001). Furthermore, highly crosslinked polyethylene was associated with lower revision rates than non-crosslinked (HR, 0.48; 95% CI, 0.29–0.79; *p* = 0.003). Conversely, hydroxyapatite-coated cementless prostheses unexpectedly demonstrated higher revision rates than non-coated (HR, 1.87; 95% CI, 1.44–2.43; *p* < 0.001), with stratified analyses indicating effect modification by bearing type. Reflecting these risks, the AOANJRR formally identified the Hintermann Series H3 prosthesis combination as having a higher-than-anticipated revision rate (10-year CPR 21.0%; 95% CI, 17.5–25.2) [11]. A Dutch register-based analysis (*n* = 1246) similarly reported a lower revision risk with fixed-bearing designs [25]. St Mart et al. (2024) reported significant survival improvements with successive implant generations in 4642 contemporary TAAs [26], but surgical learning-curve effects were inconsistent [27].

### 4.5. Strengths and Limitations

This study draws on a large, combined cohort of over 18,000 primary TAAs from three established national registries, enhancing generalisability compared with single-registry analyses. Four SwedAnkle CPR values (marked with asterisks in Table 2) were read from Kaplan–Meier curves rather than from tabulated registry data, with a reading precision of approximately ±1–2 percentage points. These approximations do not affect the cross-registry comparisons that underpin the study’s conclusions. Other limitations include: (1) descriptive analysis of aggregate data without patient-level access, precluding cross-registry multivariable adjustment; (2) non-commensurate metrics across registries; (3) SwedAnkle’s inclusion of historical implants from 1993; (4) NJR-documented under-reporting of arthrodesis and amputation, with unknown analogous under-reporting in AOANJRR and SwedAnkle [10]; (5) risk factor findings drawn exclusively from AOANJRR multivariable analyses; (6) registry-reported *p*-values not adjusted for multiple testing; and (7) wide CIs for long-term estimates (beyond 10 years) due to the diminishing number of patients at risk; (8) SwedAnkle’s explicit exclusion of isolated insert exchange from its Kaplan–Meier endpoint, creating a systematic downward bias in its reported revision rates relative to the NJR and AOANJRR (magnitude discussed in Section 4.1); and (9) incomplete geographical coverage—the three registries analysed do not represent the full international TAA population, and reported patterns may not automatically extrapolate to other registries or health systems.

## 5. Conclusions

This three-registry descriptive analysis of 18,552 primary TAAs shows that aseptic loosening is the predominant indication for revision after TAA, with infection contributing substantially in the NJR and AOANJRR (and ranking third behind insert wear/breakage in SwedAnkle). AOANJRR multivariable analyses (osteoarthritis sub-cohort for age, sex, BMI, and ASA; all-diagnoses primary cohort for surgical era) identified younger age, obesity, and earlier surgical eras as statistically significant independent predictors of revision. Recent operative eras and newer implant designs were associated with improved survivorship: 10-year revision rates of approximately 10% have been reported in the contemporary NJR cohort, and the all-eras AOANJRR osteoarthritis sub-cohort 10-year CPR was 13.5% (95% CI, 12.1–15.1%), positioned between the NJR contemporary and historical Swedish estimates. While absolute revision rates differ substantially between registries (10–26% at 10 years), and the methodological heterogeneity precludes direct cross-registry comparison, these findings show consistent qualitative patterns across registries (i.e., aseptic loosening as the predominant cause of revision, and improving survivorship with newer implant generations) that may inform preoperative counselling, implant selection, and ongoing efforts toward registry harmonisation.

## Figures and Tables

**Table 1:** Registry characteristics and metric definitions. Direct numerical comparison of the metrics shown is not appropriate; see footnote and Section 4.1 for differences in revision definitions, follow-up, and reporting structure.

Characteristic	NJR (UK)	AOANJRR (Australia)	SwedAnkle (Sweden)
Report year	2025	2025	2024
Data coverage	Jan 2010–Dec 2024	2006–Dec 2024	1993–2024
Total primary TAAs	11,321	5379	1852
Reported age measure ^1^	69 (IQR 62–75)	68 (most common age group 65–74)	~64 years (2024 cohort only)
Female (%)	4458 (39.4)	2041 (38)	~41% (2024 only)
Primary diagnosis	Mixed (OA dominant)	OA cohort available	OA dominant
Revision metric	Incidence rate per 100 prosthesis-years	% of all revisions; Cox HRs	% of all revisions; KM survivorship
Minor exchanges counted?	Yes	Yes	No (meniscus exchange excluded from KM endpoint)
Revision endpoint includes	Component addition/removal/modification, debridement, antibiotics, and implant retention with exchange, conversion to arthrodesis, removal of prosthesis, and amputation. Note: Arthrodesis and amputation may be under-reported [10].	Insert exchange, metal component revision, conversion to arthrodesis, and removal of prosthesis [11].	Metal component exchange or removal, and arthrodesis. Isolated meniscus exchange is tracked but excluded from KM survivorship endpoint [12,14].

^1^ Direct numerical comparison of the rows is not appropriate. The three registries differ in revision definition (insert exchange included in NJR and AOANJRR, excluded from the SwedAnkle survival endpoint), implant generations covered, follow-up windows, and statistical structure; cross-registry comparisons throughout the manuscript are therefore descriptive only. Age and sex metrics are not directly commensurable: NJR reports median (IQR); AOANJRR reports the most common age group (65–74 years) and median; SwedAnkle figures (~64 years; ~41% female) reflect the 2024 calendar-year cohort only (*n* = 136 primary procedures in that year; SwedAnkle Annual Report 2024).

**Table 2:** CPR at standard time points. Direct numerical comparison of the CPR values across registries is not appropriate: the three registries differ in revision definitions (notably the SwedAnkle exclusion of isolated insert exchange from the survival endpoint), implant generations covered, and follow-up windows (see Section 4.1); all cross-registry comparisons in this table are descriptive.

Registry	Cohort/Era	No.	1 Year	3 Years	5 Years	7 Years	10 Years	14 Years	15 Years	20 Years	Method
NJR (UK)	All primary ankle replacements	11,321	0.73	2.97	5.13	6.89	9.54 ^†^	11.47	NR	NR	Direct KM
AOANJRR (Australia)	Primary TAA, 2015–2024	3858	1.5	3.8	5.1	6.4	NR	NR	NR	NR	Direct, period-specific KM
AOANJRR (Australia)	Primary TAA, pre-2015	1521	2.8	7.2	10.7	12.9	17.0	NR	20.5	NR	Direct, period-specific KM
SwedAnkle (Sweden)	Undén et al. (2020) [14] sub-cohort (prostheses to 2016)	1226	NR	NR	15.0 *	NR	26.0 *	NR	37.0 *	42.0 *	Derived *

Abbreviations: CPR = cumulative per cent revision; KM = Kaplan–Meier; NR = not reported; TAA = total ankle arthroplasty. * The marked SwedAnkle values at 5, 10, 15, and 20 years were read from the Kaplan–Meier curves published by Undén et al. (2020) [14] for their cohort of 1226 prostheses implanted through 2016 and converted using CPR = 100 × (1 − survival fraction); see Section 2.1 for the extraction procedure and ±1–2 percentage point precision. The SwedAnkle cohort in this table (*n* = 1226) is smaller than the full registry of 1852 primary TAAs. ^†^ NJR 10-year CPR 9.54% (95% CI, 8.75–10.39); 14-year CPR 11.47% (95% CI, 10.36–12.68) (NJR Table 3.A3 [10]). Note: the NJR explicitly acknowledges under-capture of conversion to arthrodesis and amputation, which would otherwise increase the reported revision rate.

**Table 3:** Indications for revision. Descriptive comparisons across registries.

Indication	NJR (Rate per 100 Prosthesis-Years)	AOANJRR (*n*, %)	SwedAnkle (*n*, %)
Aseptic loosening	0.43	140 (33.0)	169 (47.6)
Infection	0.27	61 (14.4)	34 (9.6)
Lysis	0.22	40 (9.4)	NR *
Implant breakage (insert)	NR	38 (9.0)	41 (11.5)
Instability	NR	34 (8.0)	17 (4.8)
Malalignment	0.19	7 (1.7)	22 (6.2)

NR = not reported. * Lysis-driven failures captured under aseptic loosening in SwedAnkle. NJR: incidence rates per 100 prosthesis-years (Table 3.A5 [10]; indications not mutually exclusive). AOANJRR: 424 first revisions of known primaries (Table A12). SwedAnkle: 355 cumulative revisions, 1993–2024 (SwedAnkle Annual Report, Table 8 [12]). Direct quantitative comparison of the columns is precluded by differences in metric structure (rates per 100 prosthesis-years vs. percentages of all revisions), endpoint definitions (SwedAnkle excludes isolated insert exchange from its survival endpoint), implant generations covered, and follow-up windows; all cross-registry comparisons in this table are descriptive only, identifying qualitative consistencies rather than numerical equivalence (see Section 4.1).

**Table 4:** Type of revision surgery performed (AOANJRR, *n* = 424).

Type of Revision	Number (*n*)	Percentage (%)
Insert-only	190	44.8
Tibial/talar	82	19.3
Arthrodesis	49	11.6
Tibial-only	39	9.2
Talar-only	30	7.1
Cement spacer	20	4.7
Removal of prosthesis	7	1.7
Minor components	7	1.7
**Total**	**424**	**100.0**

Source: AOANJRR 2025 [11] Ankle Supplementary Report, Table A13. Percentages may not sum to 100 because of rounding.

**Table 5:** CPR by age group (AOANJRR, OA cohort).

Age Group	5-Year CPR % (95% CI)	7-Year CPR % (95% CI)	10-Year CPR % (95% CI)
<55 years	10.5 (7.3–14.8)	11.6 (8.2–16.4)	14.5 (10.4–20.0)
55–64 years	9.8 (8.1–11.9)	13.2 (11.1–15.8)	19.1 (16.3–22.4)
65–74 years	6.6 (5.4–8.0)	8.3 (6.9–10.0)	11.8 (9.8–14.1)
≥75 years	5.3 (3.9–7.2)	5.9 (4.3–7.9)	7.2 (5.2–9.9)

Registry-derived KM estimates with 95% CI in parentheses. Source: AOANJRR 2025 Ankle Supplementary Report, OA cohort.

**Table 6:** Age–sex pattern: 7-year CPR (NJR, all cases).

Age Group	Female 7-Year CPR % (95% CI)	Male 7-Year CPR % (95% CI)
<65 years	11.52 (9.73–13.61)	8.53 (7.16–10.14)
65–74 years	6.75 (5.40–8.42)	6.52 (5.45–7.79)
≥75 years	2.05 (1.26–3.33)	3.71 (2.71–5.07)

Calculated 7-year KM estimates, with 95% CI in parentheses. Source: NJR 22nd Annual Report 2025, Table 3.A3 [10]. NJR-derived cumulative per cent revision figures should be interpreted as lower bounds: linked NJR–NHS Digital analysis shows that approximately one-third of true failures are not captured by the NJR alone [17]; the true 7-year age-sex CPR values are therefore plausibly higher than shown.

**Table 7:** HR for BMI and ASA score (AOANJRR, OA cohort).

Risk Factor	Comparison	HR	95% CI	*p*-Value
BMI (obesity)	≥30 vs. <30 kg/m^2^	1.52	1.06–2.19	0.023
ASA score	ASA 3 vs. ASA 1 ^‡^	1.12	0.65–1.96	0.678
ASA score	ASA 2 vs. ASA 1 ^‡^	1.28	0.77–2.13	0.332

^‡^ ASA 1 is the reference category. Source: AOANJRR 2025 Ankle Supplementary Report, Cox models, OA cohort.

**Table 8:** Summary of all HR (AOANJRR Cox proportional hazards models, OA cohort).

Risk Factor	HR	95% CI	*p*-Value
Age (<55 vs. ≥75 years)	2.00	1.30–3.07	0.001
Surgical era (pre-2015 vs. 2015–2024)	1.91	1.53–2.38	<0.001
BMI (≥30 vs. <30 kg/m^2^)	1.52	1.06–2.19	0.023
Sex (male vs. female)	1.22	0.99–1.50	0.063
ASA score (ASA 3 vs. ASA 1) ^‡^	1.12	0.65–1.96	0.678
ASA score (ASA 2 vs. ASA 1) ^‡^	1.28	0.77–2.13	0.332

^‡^ ASA 1 is the reference. Source: AOANJRR 2025 Ankle Supplementary Report. Age, sex, BMI, and ASA HRs are from the osteoarthritis sub-cohort (*n* = 5079; Tables A16–A19, Cox models adjusted for age/sex). The surgical-era HR is from the all-diagnoses primary cohort (*n* = 5379; Table A14 [11], Figure A7; adjusted for age/sex).

## Data Availability

No new data were created or analyzed in this study. All data analysed are publicly available in the respective national registry annual reports, which are cited in the References section: the NJR 22nd Annual Report 2025 (United Kingdom), the AOANJRR 2025 Ankle Supplementary Report (Australia), and the SwedAnkle Annual Report 2024 (Sweden).
